# Multicenter assessment of sedation and delirium practices in the intensive care units in Poland - is this common practice in Eastern Europe?

**DOI:** 10.1186/s12871-017-0415-2

**Published:** 2017-09-02

**Authors:** Katarzyna Kotfis, Małgorzata Zegan-Barańska, Maciej Żukowski, Krzysztof Kusza, Mariusz Kaczmarczyk, E. Wesley Ely

**Affiliations:** 1 Department of Anaesthesiology, Intensive Therapy and Acute Intoxications, Pomeranian Medical University, Szczecin, Poland; 2 Department of Anesthesiology and Intensive Therapy, Poznan University of Medical Sciences University, Poznań, Poland; 3 Department of Clinical and Molecular Biochemistry, Pomeranian Medical University, Szczecin, Poland; 4 Department of Medicine/Allergy, Pulmonary and Critical Care, Vanderbilt University School ofMedicine, Vetaran’s Affairs Geriatric Research Education Clinical Center (GRECC) for Tennessee Valley, Department of Veterans Affairs Medical Center, Tennessee Valley Healthcare System, Nashville, TN USA

**Keywords:** Sedation, ICU delirium, Guidelines, CAM-ICU

## Abstract

**Background:**

The majority of critically ill patients experience distress during their stay in the Intensive Care Unit (ICU), resulting from systemic illness, multiple interventions and environmental factors. Providing humane care should address concomitant treatment of pain, agitation and delirium. The use of sedation and approaches to ICU delirium should be monitored according to structured guidelines. However, it is unknown to what extent these concepts are followed in Eastern European countries like Poland. The aim of this study was to evaluate sedation and delirium practices in ICUs in Poland, as a representative of the Eastern European block, particularly the implementation of sedation and ICU delirium screening tools, availability of written sedation guidelines, choice of sedation and delirium treatment agents.

**Methods:**

A national postal survey was conducted in all Polish ICUs in early 2016.

**Results:**

A total of 165 responses out of 436 addressed units were received (37.8%). Out of responding ICUs delirium is monitored in only 11.9% of the units in Poland. Sedation monitoring tool is used in only 46.1% of units. Only 19.4% of ICUs have written protocols for sedation and 32.1% do not practice daily sedation interruption. The most frequently used agents for short-term sedation (<24 h) were propofol and fentanyl infusions and benzodiazepines (midazolam) and morphine for longer sedation (>24 h). The preferred agents for delirium treatment were haloperidol (77.6%), dexmedetomidine (43.6%) and quetiapine (19.4%). Close to one-third (32.7%) of respondents chose a benzodiazepine (diazepam) for ICU delirium treatment. Non-pharmacological treatment for ICU delirium was reported by only 45% of the respondents.

**Conclusions:**

A majority of Polish ICUs do not adhere to international guidelines regarding sedation and delirium practices. There continues to be inadequate use of sedation and delirium monitoring tools. High usage of benzodiazepines for sedation and ICU delirium treatment reveals persistence of non-evidence-based practice. This study should prompt further assessment of other Eastern Europe countries and help generate a collective response to update these aspects of patient safety and comfort.

## Background

Patients undergoing treatment in the intensive care unit (ICU) experience many distressing and potentially painful interventions [1]. Sedation and analgesia administered to critically ill patients to enable highly distressing therapies, such as mechanical ventilation and multiple invasive procedures, must focus on ensuring patient comfort, safety, and maximally humane care, therefore sedatives and analgesics are the most commonly administered medications in the ICU [2]. Historical approach delivering deep sedation to paralyzed patient is no longer accepted due to certain advances of ICU care, including improvement in microprocessor-controlled mechanical ventilation synchronizing with patients’ own respiratory efforts and new shorter-acting sedative and analgesic medications [3]. Concomitant to technical and pharmacological advancement the concept and recognition of the ICU Triad (pain, agitation delirium) emerged, placing patient’s brain and psychological distress at the same level of importance as physiological derangement [3]. The recognition of pain, excessive sedation, and delirium are now recognized as major problematic issues in the ICU that are associated with increased morbidity and mortality in the acute and long-term outcomes of critically ill patients.

Sedation can be used to maintain comfort for the patient and minimize agitation and anxiety. It can represent a whole spectrum of approaches from light sedation (when the patient is able to follow simple commands and is easily arousable) to deep sedation (when the patient is not able to respond to painful stimuli) [2]. Both deep sedation and the initiation of sedation within first 48 h of ICU admission is associated with increased in-hospital mortality, prolonged ICU and hospital stay, prolonged mechanical ventilation, and decreased two-year follow-up survival [4]. Current international sedation guidelines concentrate on eliminating benzodiazepines in favor of strategies based on analgesia and use of propofol or dexmedetomidine to improve clinical outcome in critically ill patients, namely decreased ICU and hospital treatment time, decreased likelihood of developing delirium and post-ICU cognitive impairment [5, 6]. Minimizing sedation, engaging strategies targeting light sedation and using validated sedation assessment tools is the goal of modern ICU treatment [2, 6–8].

Also critical illness related delirium (ICU Delirium) is not only associated with, but an independent predictor of dire consequences, including increased mortality, prolonged ICU and hospital stay, and long-term memory loss. According to different studies, ICU Delirium affects 50–80% of mechanically ventilated adult ICU patients [3]. Therefore, this form of organ brain dysfunction must be regarded as a major public health problem with significant financial consequences. The true difficulty in approaching ICU delirium is to understand that it may be either disease-associated (i.e. sepsis, heart failure, respiratory failure, pneumonia), iatrogenic (i.e. unnecessary sedation, benzodiazepine use) or related to environmental factors (i.e. sleep deprivation, immobilization, use of physical restraints). It must be underlined that only early detection of delirium with dedicated and reliable delirium assessment tools gives patients a chance to receive proper treatment to reduce the untoward outcomes related to delirium [9].

The purpose of our investigation was to evaluate sedation and delirium practices in intensive care units in Poland as a representative of the Eastern European countries. We are not assuming that other Eastern European countries are the same, but rather envisioned this investigation as a starting point to understand the regions critical care practices and to spark collection of more comprehensive data gathering. This survey attempted, therefore, to gain understanding of the practices of implementation of sedation and ICU delirium monitoring tools, availability of written sedation guidelines, choice of sedation and delirium treatment agents.

## Methods

We conducted a nationwide survey on sedation and delirium practices and their determinants in ICUs across Poland (February–March 2016). Intensivists (anesthesiologists) who are head physicians working in adult ICUs composed our target population. Altogether 436 adult ICUs in hospitals across Poland were identified and addressed, with neonatal and pediatric units excluded due to differences in analgosedation guidelines and approach. Available databases did not contain complete and reliable information on the size of target population and sampling frame. In order to perform the postal survey we prepared an address database, based on data from the National Health Fund (NFZ – Narodowy Fundusz Zdrowia). We conducted a preliminary survey among physicians working in our ICU to identify the clarity of questions and phrases. We received permission from our Investigational Review Board (IRB) to publish these anonymous survey data.

### Survey instrument construction

A team including ICU consultants (KKotfis, MZB), heads of two ICU departments (KKusza, MŻ), and a statistician (MK) was involved to construct the survey tool (the exact tool is available upon request). We identified important questionnaire items through a literature review and previous qualitative studies and the final draft of the questionnaire was prepared by the first author (KKotfis). The content validity was determined and the survey team members were asked to comment on the importance of items and clarity of phrasing included in the questionnaire. Debatable issues regarding nomenclature of problems and interpretation of the questions were also discussed as suggested by other studies [10].

The survey instrument was structured into three sections:

I. General demographic data regarding ICUs and respondents (status of the hospital, number of beds in the hospital, number of ICU beds, type of activity (medical, surgical, medico-surgical, practice settings); ICU outcome monitoring (yes/no),
II. Sedation monitoring and treatment practices (use of sedation scales, sedation protocols, reported choices for analgosedation drugs and common perceptions regarding their uses),
III. ICU Delirium monitoring and treatment practices (use of ICU Delirium scales, treatment protocols, pharmacological and non-pharmacological approach).

The questions included in the final version of the questionnaire were multiple choice; for those regarding common perceptions, participants were to indicate their preferences on a 10-point numeric scale (0–10 points). The questions referred to participants’ perceptions of their ICU everyday practice and to the patients they cared for in their ICUs. Survey instrument had undergone pretesting in the ICU of the Pomeranian Medical University with two senior ICU consultants not involved in this study. The questionnaire included a cover letter and the layout, format, attractivity, ordering of questions, respondent burden, and understanding of each question (appropriateness of vocabulary and background knowledge) was evaluated before disseminating the questionnaire.

### Survey delivery and dissemination

The survey instrument was prepared in Polish and was printed on paper. The electronic version was available, but not tested as not all e-mail addresses of ICUs and ICU heads were officially available (professional e-mail addresses were lacking). Therefore the authors decided to perform a postal survey. The survey was sent via traditional post at the beginning of February 2016, to the heads/clinical directors of all Polish adult critical care units, who were asked to complete the survey on paper. A cover letter detailing the idea and purpose of the study was provided with the survey and described the objectives of the survey, mentioned support from professional national societies (the National Anesthesiology and Intensive Therapy Consultant in Poland) and assured participants of the confidentiality of their responses and contextualized the survey in terms of their everyday practice. A pre-labelled, stamped envelope was provided to respondents to return the paper survey questionnaire. To increase the response rate the units were followed up with a telephone call in mid-March 2016.

### Ethical concerns

The Bioethical Committee of the Pomeranian Medical University approved our research protocol and waived the need for written informed consent for the study (KB-0012/506/12/16). Participation in the survey was anonymous, voluntary and non-interventional. The cover letter included information that confidentiality of the responses provided is guaranteed. The respondents received a questionnaire by post and were instructed to return it to the address provided on a stamped envelope.

### Statistical analysis

To describe practices and demographic characteristics, we used descriptive statistics including mean values and standard deviations for continuous variables, and frequencies and percentages for categorical variables. We used Pearson’s chi-squared test (*χ* ^2^) to analyse categorical data differences. Statistical significance was set at *p* < 0.05.

## Results

### Response rate and demographic data

The overall response rate was 37.8% (165/436 participating ICUs). The respondents were specialists in anesthesia and intensive care who are heads of the intensive care units with a mean age of 52 years and a mean of 26 years of ICU practice. Majority of the responses came from non-academic centers - 84.4% (140/165), mixed medico-surgical units - 73.3% (121/165), moderate in size (5–8 beds) – 53.3% (88/165) with relatively low volumes of ICU admissions (<250 per year) – 67.7% (111/165). Reported characteristics showed that majority of patients (>76%) are mechanically ventilated – 79.4% (131/165). On the other hand majority of units - 91.5% (151/165) declare the use of neuromuscular blocking agents in less than 5% of patients. The data regarding hospital characteristics are depicted in Table 1.

**Table 1:** Hospital and respondents demographic data

Hospital demographic data	Total (*n* = 165)
Hospital type, % (*n*)	
Non-academic (Regional, General)	84.8 (140)
Academic (University, Institute)	15.2 (25)
ICU type, % (*n*)	
Medical	11.5 (19)
Surgical	15.2 (25)
Medico-surgical	73.3 (121)
Number of ICU beds, % (*n*)	
0 to 4 beds	21.2 (35)
5 to 8 beds	53.3 (88)
9 to 12 beds	13.9 (23)
>12 beds	11.5 (19)
Number of ICU admissions per year, % (*n*)	
<250 admissions	67.7 (111)
250 to 500 admissions	26.8 (44)
500 to 750 admissions	2.4 (4)
750 to 1000 admissions	1.2 (2)
>1000 admissions	1.8 (3)
ICU proportion of mechanically ventilated patients, % (n)	
0 to 25%	1.2 (2)
26 to 50%	3.6 (6)
51 to 75%	15.8 (26)
76 to 100%	79.4 (131)
Patients requiring muscle relaxants, % (*n*)	
0 to 5%	91.5 (151)
6 to 10%	4.9 (8)
11 to 15%	1.2 (2)
15 to 20%	2.4 (4)
Daily physiotherapy services on ICU, % (*n*)	
Yes	73.3 (121)
No	26.7 (44)
Respondents demographic data	Total (*n* = 165)
Age (mean ± SD)	52.02 ± 6.18
Number of years in ICU practice (mean ± SD)	26.2 ± 6.16
ICU Outcome monitoring	Total (*n* = 165)
ICU follow-up monitoring, % (*n*)	
Yes	23.1 (38)
No	76.9 (127)

*n* number of ICU participating in the survey, *SD* standard deviation

### Sedation monitoring and practices

#### Sedation monitoring

Only 46.1% (76/165) of respondent**s** reported using sedation scales to assess sedation practice in ICU. The most frequently chosen scales were Ramsay scale - 22.4% (37/165) and Richmond Agitation Sedation Scale - 18.8% (31/165). Sedation protocol implementation was reported in one fifth of the ICUs - 19.4% (32/165) and daily sedation interruption (DSI) was performed by only 32.1% (53/165) of units. Quality monitoring with regular sedation practices audit was declared by only 13.9% (23/165) of the participating units. Almost 51% of respondents (84/165) declared change of sedation during weaning. Action time had more influence on choice of sedative than price as scored on a 0–10 point scale (action time – mean 6.39 ± 2.77 SD, price - mean 4.06 ± 3.14 SD). All of the available data is summarized in Table 2.

**Table 2:** Sedation practices in Polish ICUs

Sedation data	Total (*n* = 165)
Sedation monitoring, % (*n*)	
No	53.9 (89)
Yes	46.1 (76)
Ramsay	22.4 (37)
RASS	18.8 (31)
BIS	1.8 (3)
Other	3.0 (5)
Sedation protocol, % (*n*)	
Yes	19.4 (32)
No	80.6 (133)
ICUs declaring daily sedation interruption (DSI), % (*n*)	
Yes	32.1 (53)
No	67.9 (112)
ICUs declaring regular sedation audit, % (*n*)	
Yes	13.9 (23)
No	86.1 (142)
ICUs declaring change of sedation during weaning, % (*n*)	
Yes	50.91% (84)
No	49.09% (81)
Choice for sedation vs price (0–10 points scale), mean ± SD	4.06 ± 3.14
Choice for sedation vs action time (0–10 point scale), mean ± SD	6.39 ± 2.77

*n* number of ICUs participating in the survey, *ICU* intensive care unit, *DSI* daily sedation interruption, *RASS* Richmond Agitation Sedation Scale, *BIS* Bispectral Index, *SD* standard deviation

#### Sedation choices for less than 24 h

**Fig. 1:**
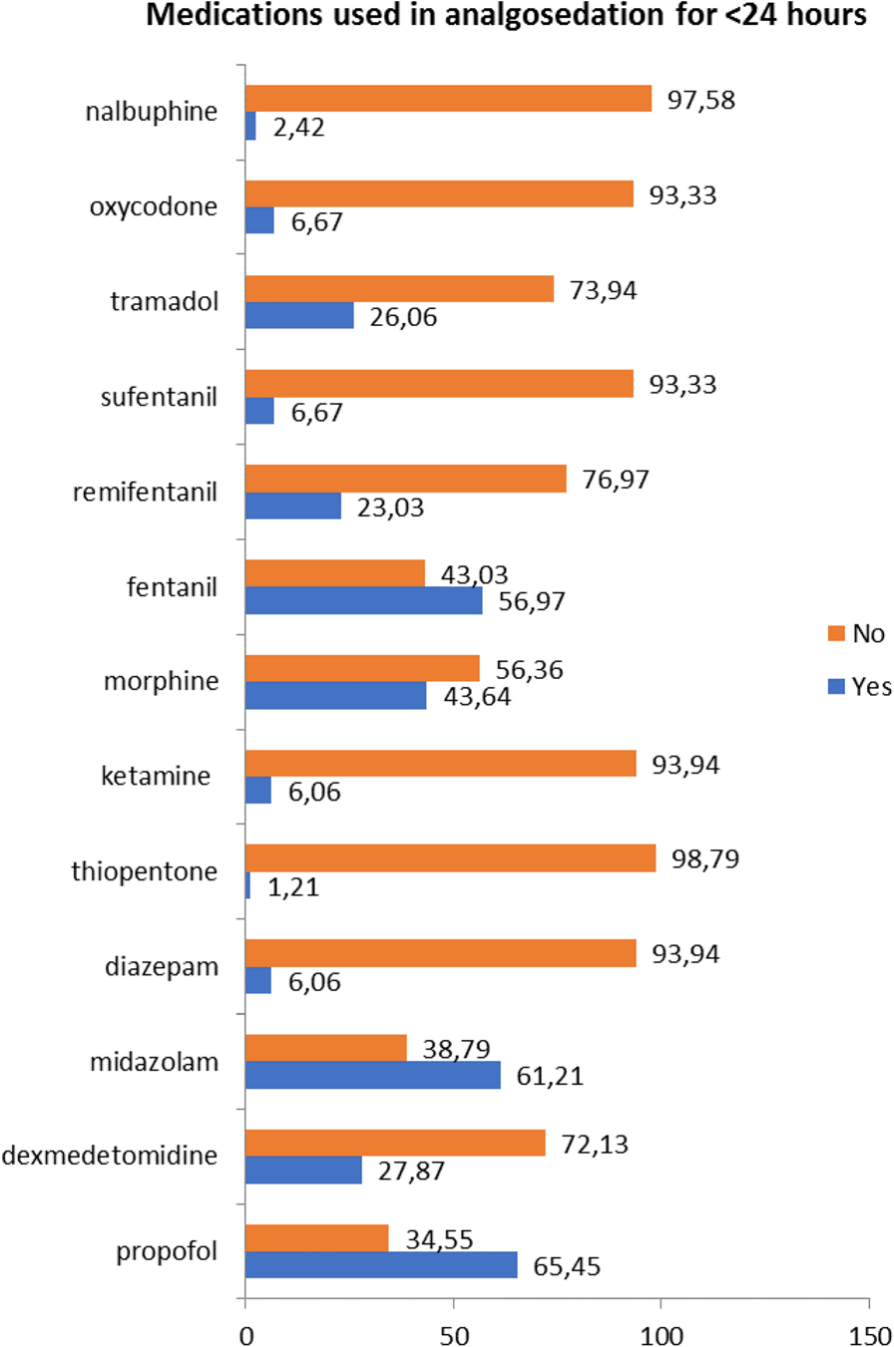
Choice of agents used for sedation for <24 h (numbers indicate percentages)

As shown in Fig. 1, the primary opioid chosen by the respondents for short term sedation was fentanyl (56.97%), followed by morphine (43.64%), tramadol (26.06%) and remifentanil (23.03%). The choices of sedatives included almost equal results for propofol (65.45%) and midazolam (61.21%). Dexmedetomidine was indicated as a choice for short-term sedation by 27.87% of respondents. Nalbuphine or oxycodone were scarcely used: 2.42% and 6.67% respectively, all respondents declared no use of lorazepam or methadone for short term sedation.

#### Sedation choices for more than 24 h

**Fig. 2:**
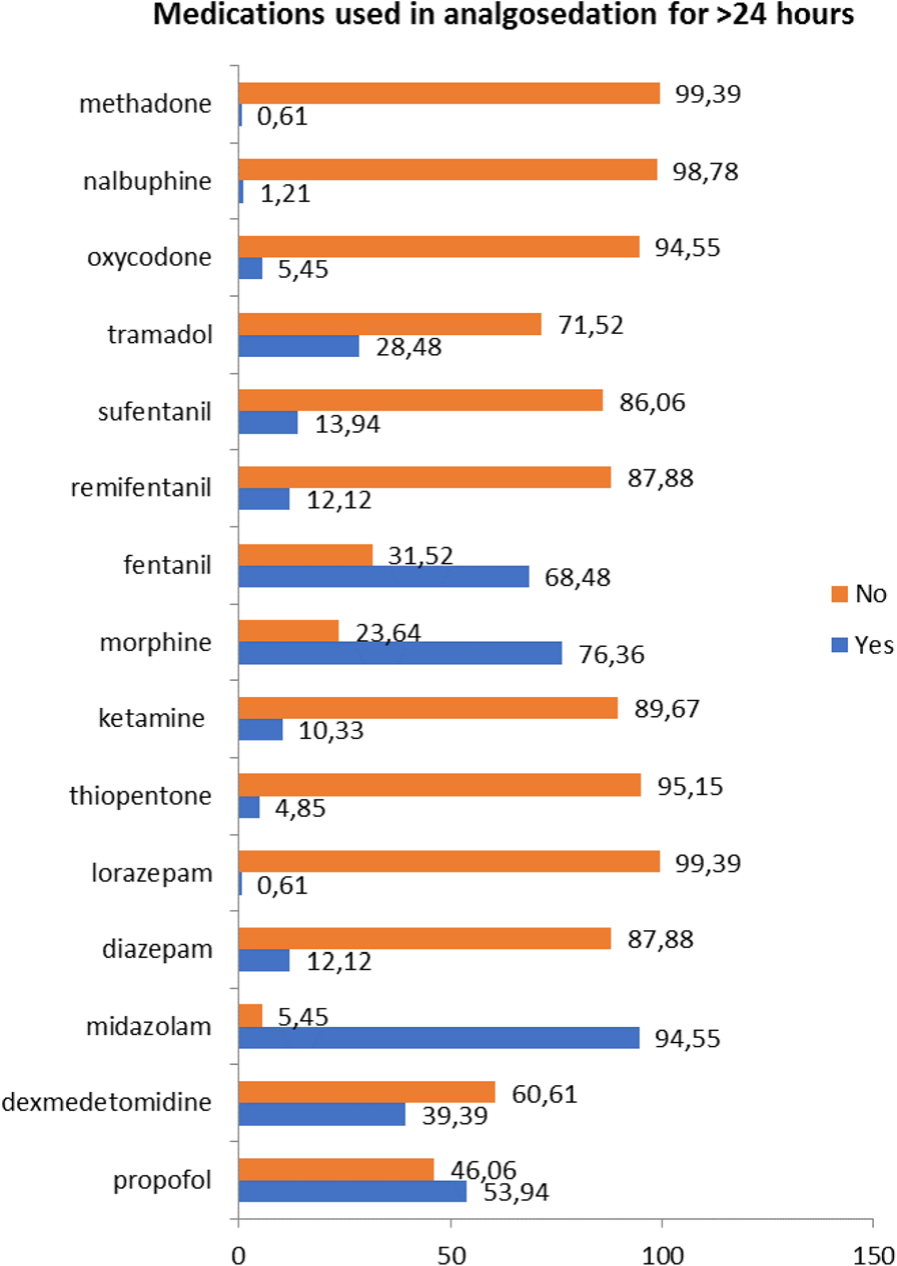
Choice of agents used for sedation for >24 h (numbers indicate percentages)

The primary opioid chosen by the respondents for long-term sedation (>24 h) was morphine (76.36%), followed by fentanyl (68.48%) and tramadol (28.48%) and almost equal responses for sufentanil (13.94%) and remifentanil (12.12%). The choices for prolonged (>24 h) sedation included predominant answers for midazolam (94.55%). Similar percentages were obtained for propofol (53.94%) and dexmedetomidine (39.39%). All the data is visible in Fig. 2.

### ICU delirium monitoring and practices in Poland

Only one-tenth of the respondents declare monitoring of the ICU delirium, namely 10.9% (18/165). In the units that declare ICU delirium monitoring, the most frequently used approach reported was based on applying the ICD 10 criteria (5.45%), followed by CAM-ICU tool use (3.03%) or applying DSM-IV criteria (2.42%). None of the units declared the use of ICDSC to identify delirium in their patients. Only one-tenth of the respondents declared having an ICU delirium treatment protocol - 10.3% (17/165). The choice of therapies used to treat a diagnosed ICU delirium included both pharmacological and non-pharmacological approach, with the latter reported in 44.8% of cases (74/165). It is visible that only 3.64% (6/165) respondents declare ICU delirium not to be a significant medical problem, with the mean response on a 0-to-10 scale being 5.25. This data is summarized in Table 3.

**Table 3:** ICU Delirium practices in Polish ICUs

ICU Delirium data	Total (*n* = 165)
ICU Delirium monitoring, % (*n*)	
Yes	10.9 (18)
CAM-ICU	3.03 (5)
ICDSC	0 (0)
DSM-IV	2.42 (4)
ICD 10	5.45 (9)
No	89.1 (147)
ICU Delirium treatment protocol, % (*n*)	
Yes	10.3 (17)
No	89.7 (148)
Non-pharmacological approach in ICU Delirium, % (*n*)	
Yes	44.8 (74)
No	55.2 (91)
ICU delirium regarded as medical problem (0–10 scale)	
Mean score ± SD	5.25 ± 2.59
0 – not a problem, % (*n*)	4.24 (7/165)
1 – major medical problem, % (*n*)	5.45 (9/165)

*ICU* intensive care unit, *CAM-ICU* Confusion Assessment Method for ICU, *ICDSC* Intensive Care Delirium Screening Checklist, *DSM IV* Diagnostic and Statistical Manual of Mental Disorders, *ICD 10* International Statistical Classification of Diseases and Related Health Problems

**Fig. 3:**
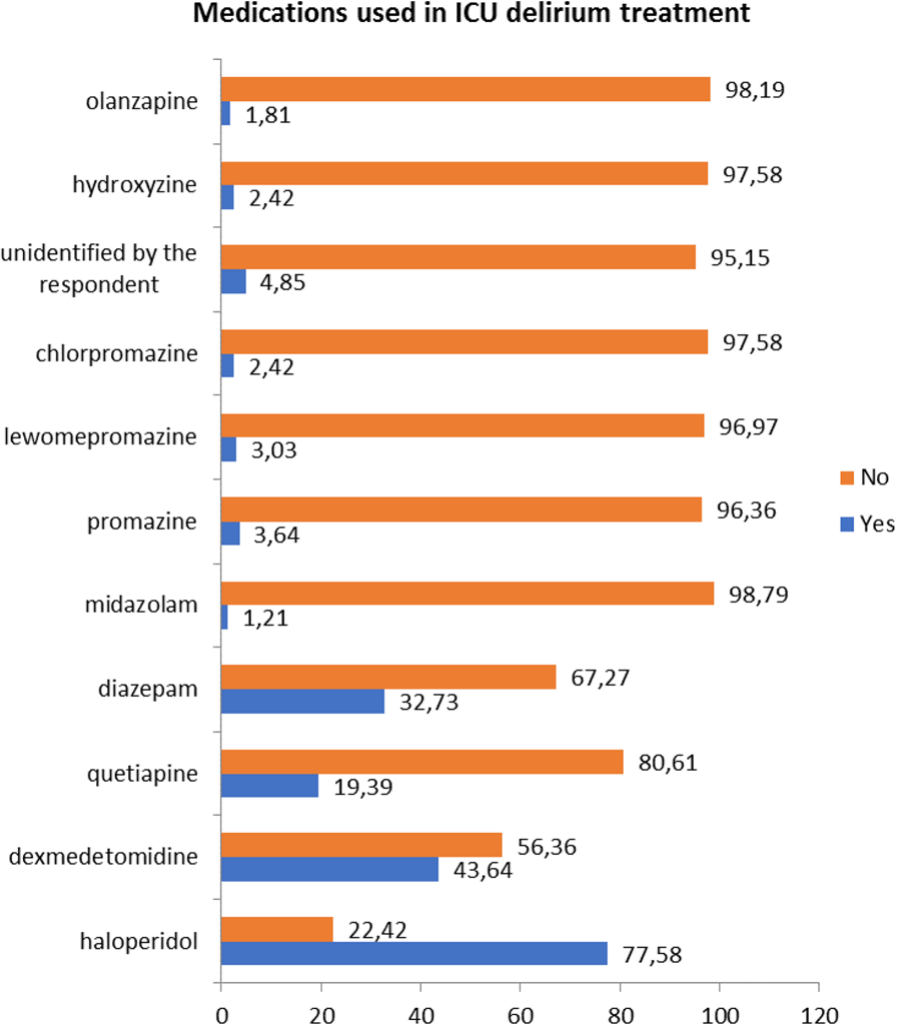
Choice of agents used for ICU Delirium treatment (numbers indicate percentages)

Figure 3 shows medications chosen to treat ICU delirium. When analyzing the pharmacological approach to ICU Delirium it is visible that the majority of ICU heads chose haloperidol (77.58%), dexmedetomidine (43.64%) and quetiapine (19.39%), but also one-third (32.73%) chose a benzodiazepine (namely diazepam). The remaining medications included midazolam, phenothiazine antipsychotics (promazine, levomepromazine and chlorpromazine), hydroxyzine and olanzapine chosen by less than 5% of respondents.

**Fig. 4:**
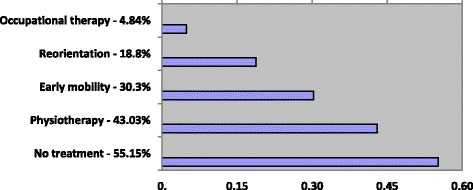
Non-pharmacological treatment for ICU Delirium in Polish ICUs

An analysis of non-pharmacological approach towards ICU delirium treatment shows that mainstay was physiotherapy declared by 43.03% of units and early mobility reported by 30.03% of responding ICUs. This data is followed by reorientation implemented in 18.8% of units and occupational therapy available to 4.84% of patients. The data regarding non-pharmacological ICU Delirium approach is depicted in Fig. 4.

An analysis showing data from academic and non-academic centers is shown in Table 4. It is visible that the academic ICUs screened for delirium and used non-pharmacological measures in the treatment of ICU delirium more often than non-academic centers.

**Table 4:** Data regarding academic and non-academic centers

Data	Academic (*n* = 25)	Non-academic (*n* = 140)	p
ICU type, % (*n*)			
Medical	16 (4)	11 (15)	0.44
Surgical	32 (8)	12 (17)	<0.05
Medico-surgical	52 (13)	77 (108)	<0.05
Number of ICU admissions per year, % (*n*)			
<250 admissions	48 (12)	70.7 (99)	0.175
250 to 500 admissions	40 (10)	25 (35)	
>500 admissions	12 (3)	4.3 (6)	
ICU proportion of mechanically ventilated patients, % (*n*)			
0 to 50%	4 (1)	5 (7)	0.862
51 to 75%	20 (5)	15 (21)	
76 to 100%	76 (19)	80 (112)	
Daily physiotherapy services on ICU, % (*n*)	84 (21)	71.4 (100)	0.19
ICU Outcome monitoring, % (*n*)	28 (7)	22.1 (31)	0.521
ICU sedation monitoring, % (*n*)	56 (14)	44.3 (62)	<0.05
ICU sedation protocol, % (*n*)	16 (4)	20 (28)	0.641
Daily sedation interruption, % (*n*)	32 (8)	32.1 (45)	0.988
ICU Sedation audit, % (*n*)	20 (5)	12.9 (18)	0.342
ICU delirium screening, % (*n*)	24 (6)	8.6 (12)	<0.05
ICU delirium treatment protocol, % (*n*)	20 (5)	8.6 (12)	0.08
Pharmacological ICU delirium treatment choice, % (*n*)			
Haloperidol	72 (18)	78.6 (110)	0.468
Dexmedetomidine	72 (18)	38.6 (54)	<0.05
Quetiapine	36 (9)	16.4 (23)	<0.05
Diazepam	12 (3)	36.4 (51)	<0.05
Non-pharmacological ICU delirium treatment % (*n*)			
Physiotherapy	72 (18)	37.9 (53)	<0.05
Early mobility	60 (15)	25 (35)	<0.05
Reorientation	40 (10)	15 (21)	<0.05
Occupational therapy	12 (3)	3.57 (3)	0.07
No treatment	24 (6)	48.57 (68)	<0.05

*ICU* intensive care unit, *n* number of patients

## Discussion

Eastern European countries have, to our knowledge, not embraced current guidelines in terms of sedation and delirium monitoring, and this study conducted in Poland represents a first step in demonstrating this driving unmet need for the region. Only 1 in 5 ICUs even have a sedation protocol and only 1 in 10 monitor for delirium, the most common form of brain organ dysfunction in critical illness. These survey data call into question the fact that in 2017 no official Polish recommendations exist regarding sedation practices and the national health authority has not weighted in at all regarding implementation of myriad data that comprise the SCCM’s Pain, Agitation, and Delirium (PAD) guidelines [5].

Identification of barriers potentially impairing adherence to international guidelines may be the first step in improvement. One of the identifiable problems may be the language barrier, therefore the clinical tools (RASS, CAM-ICU, CPOT and BPS) have recently been translated into Polish after official permission was obtained from the authors of these tools [9, 11]. Looking at other Eastern European countries, it must be stressed that while the CAM-ICU is available in Czech, Serbian, Russian and Polish, there is work to be done for the other Eastern European countries for all the tools. Looking back 3 years ago, the scales available for Polish intensivists to monitor brain function were limited, but after translation of the CAM-ICU training manual by Kotfis et al. in November 2015, the manual is available for general use as posted on http://icudelirium.org and http://proicu.pl websites and was part of multiple courses (i.e. Committee for Continuing Education in Anesthesiology, CCEA) recently performed in Poland by the European Society of Anesthesiology.

When analyzing the results of this study its problems and concerns must be addressed first. A definite weakness of this study is a low participant response rate – 37.8%. The response rate in similar studies in other parts of Europe or world was much higher - Richard-Belle et al. showed a 91.1% response rate, Reschreiter et al. reported a total of 192 responses out of 302 addressed units (63.5%) [12, 13]. This phenomenon can be discussed in a variety of ways, as lack of interest, lack of time or work overload, therefore care must be taken in encouraging intensivists in Poland to participate in practice assessment and monitoring in the future in order to improve quality of care over most dependent patients. Low response rate is not satisfactory and must be regarded as a limitation of this study, but due to the fact that there is no data regarding this subject in Poland the aim of this study was to reveal an unmet need. Moreover we must assume some degree of a responder bias, as it is a known phenomenon that studies relying on self-report regarding certain clinical practices overestimate the use of evidence-based medicine as compared with real life practices. Therefore the actual frequency of use for both sedation scales and ICU delirium monitoring may be even lower than shown in our analysis and reported by ICU clinical leads. It must be underlined that we cannot provide information about the actual practices in Poland as this was a study based on a survey and not a point prevalence study. A way of overcoming this issue may be a point prevalence study as performed by Richards-Belle et al. [12] and shall be undertaken in the future by the authors.

Comparing our results to those of other researchers is also important.We reported a low, 19.4% availability rate of sedation protocols use in Polish ICUs and 46.1% rate of sedation level assessment. Sneyers et al. also reported that analgo-sedation protocols were available only to 31% of the respondents, but majority (93%) reported using sedation scales to assess the level of sedation [10]. According to Patel et al. protocolized sedation management is more common than delirium management as sedation protocol is used by only 70% of units, with a specific sedation scale used by 88% of respondents (26% used RASS, 37% used Ramsay scale) [14].

Our data shows that benzodiazepines are the mainstay of sedation in Poland, thus there is much room for improvement. Previous surveys conducted in different parts of the world (USA, UK, Australia/New Zealand, Europe) indicate a shift in sedating patients in critical care away from benzodiazepines and towards propofol [12, 15–22].

Data regarding delirium show that Polish intensivists regard it as a moderately important problem (mean score 5.25 ± 2.59 rating on a 0–10 point scale), yet only 11% of Polish intensivists screen for delirium. Moreover, hypoactive type of delirium, which is common and associated with adverse outcome, can only be diagnosed with dedicated tools [23, 24]. Without prompt identification of ICU Delirium, comprehensive approaches (e.g., environmental modifications including lighting, diurnal cycle restoration, eye glasses and hearing aid use, mobilization, and removal of untoward psychoactive drug use) will remain underutilized. According to a survey performed by Ely et al. in year 2001, majority of healthcare practitioners believed delirium was a prevalent problem, but few had protocols for managing delirium [25]. Ely et al. reported that 92% of intensivists regarded delirium as a significant or very serious problem in the intensive care unit, only 40% reported routinely screening for delirium, 16% indicated using a specific delirium screening tool. Patel et al. who performed a follow-up study to the previous one, but in years 2006–2007, have shown that 5 years later half of the respondents screen for delirium, but only one third use a specific screening tool with a 3-fold higher rate (33% vs. 12%, *p* < 0.0001) [14]. Both our study and Patel et al. show that, despite availability of validated screening tools (CAM-ICU and ICDSC) the use of validated delirium screening tools remains infrequent.

Data regarding delirium treatment shows that majority of intensivists choose haloperidol (77.58%), followed by dexmedetomidine (43.64%) and quetiapine (19.39%). A worrisome factor remains in the reported use of diazepam for ICU delirium treatment (32.73%) as this agent should be reserved for anxiety or alcohol-withdrawal delirium. According to Patel et al. 86% of respondents reported treating delirium with haloperidol and nearly 40% reported using an addition of an atypical antipsychotic. In our study the non-pharmacological approach was reported in only 44.8% of cases (74/165) - 43.03% of units declare the use of physiotherapy, 30.03% - early mobility, 18.8% - reorientation and 4.84% - occupational therapy.

When analyzing academic and non-academic centers it must be underlined that although the numbers were low (only 25 academic ICUs vs 140 non-academic ICUs) it is clearly visible that the intensivists working in academic centers use scales for sedation and screen for delirium more often than their non-academic counterparts. Moreover the use of non-pharmacological measures as well as adequate pharmacological approach in the treatment of ICU delirium is visible more often in academic centers. This fact may be due to a higher awareness and education about ICU delirium among the ICU staff working in university hospitals, but also may indirectly indicate a need for better education of intensivists in non-academic ICUs.

The fact that clinicians say that they are aware of this problem of ICU delirium and yet are not mandating patient monitoring and treatment as part of routine care is worrisome. This indicates that more education and a change of culture is warranted. ICU Liberation concepts can become part of the “new way” of patient management, and this was a focus of the SCCM’s ICU Liberation Collaborative for the past two years in the US [26]. Indeed, even easy to use tools and mnemonics for how to respond clinically when a patient is diagnosed with delirium can be taught and incorporated into daily ICU care (e.g., the Dr. DRE mnemonic – **D**isease **r**emediation, **D**rug **R**emoval, **E**nvironmental modifications).

To make the results of this study more meaningful in providing better outcome for ICU patients in Poland we propose a set actions to change practices: 1. better education of intensivists, 2. introduction of national delirium and sedation guidelines (i.e. from the Polish Society for Anesthesiology and Intensive Care) and 3. the use of delirium screening and sedation assessment scales translated into Polish and validated in Polish. It must be underlined that the goal of reducing mortality and improving quality of ICU care in Poland can only be achieved with an active participation of the health authorities.

## Conclusions

First, the majority of intensive care units in Poland still do not monitor sedation level or the presence of delirium in critically ill patients. Second, the majority of units have not introduced written sedation or ICU delirium treatment guidelines. Third, the majority of the ICUs in Poland do not embrace non-pharmacological approach to ICU delirium treatment. Unfortunately, the mainstay of sedation choices and ICU delirium treatment in Poland remains the use of benzodiazepines. It must be underlined that compliance with international sedation and delirium guidelines and recommendations in Polish ICUs is poor, and that this is likely the case in other Eastern European countries. This study should be regarded as an initial step in providing a call for action to change practices in a large part of the European continent and incorporate tools and guidelines that are otherwise being embraced globally because of the sound existing evidence related to their role in improving patient safety and comfort.

## Abbreviations

BPS: Behavioral pain scale
CAM-ICU: Confusion assessment method for intensive care unit
CCEA: Committee for continuing education in anaesthesiology
CPOT: Critical care pain observation tool
DSI: Daily sedation interruption
ICU: Intensive care unit
PAD: Pain, agitation, delirium guidelines
RASS: Richmond agitation sedation scale
